# Human infrapatellar fat pad-derived stem cells express the pericyte marker 3G5 and show enhanced chondrogenesis after expansion in fibroblast growth factor-2

**DOI:** 10.1186/ar2448

**Published:** 2008-07-03

**Authors:** Wasim S Khan, Simon R Tew, Adetola B Adesida, Timothy E Hardingham

**Affiliations:** 1 United Kingdom Centre for Tissue Engineering at the Wellcome Trust Centre for Cell Matrix Research, Faculty of Life Sciences, Michael Smith Building, University of Manchester, Oxford Road, Manchester, M13 9PT, UK

## Abstract

**Introduction:**

Infrapatellar fat pad (IPFP) is a possible source of stem cells for the repair of articular cartilage defects. In this study, adherent proliferative cells were isolated from digests of IPFP tissue. The effects of the expansion of these cells in fibroblast growth factor-2 (FGF-2) were tested on their proliferation, characterisation, and chondrogenic potential.

**Methods:**

IPFP tissue was obtained from six patients undergoing total knee replacement, and sections were stained with 3G5, alpha smooth muscle actin, and von Willebrand factor to identify different cell types in the vasculature. Cells were isolated from IPFP, and both mixed populations and clonal lines derived from them were characterised for cell surface epitopes, including 3G5. Cells were expanded with and without FGF-2 and were tested for chondrogenic differentiation in cell aggregate cultures.

**Results:**

3G5-positive cells were present in perivascular regions in tissue sections of the IPFP, and proliferative adherent cells isolated from the IPFP were also 3G5-positive. However, 3G5 expression was on only a small proportion of cells in all populations and at all passages, including the clonally expanded cells. The cells showed cell surface epitope expression similar to adult stem cells. They stained strongly for CD13, CD29, CD44, CD90, and CD105 and were negative for CD34 and CD56 but were also negative for LNGFR (low-affinity nerve growth factor receptor) and STRO1. The IPFP-derived cells showed chondrogenic differentiation in cell aggregate cultures, and prior expansion with FGF-2 enhanced chondrogenesis. Expansion in FGF-2 resulted in greater downregulation of many cartilage-associated genes, but on subsequent chondrogenic differentiation, they showed stronger upregulation of these genes and this resulted in greater matrix production per cell.

**Conclusion:**

These results show that these cells express mesenchymal stem cell markers, but further work is needed to determine the true origin of these cells. These results suggest that the expansion of these cells with FGF-2 has important consequences for facilitating their chondrogenic differentiation.

## Introduction

Cartilage is frequently damaged by trauma and in disease and has a poor ability to heal. Cartilage defects that extend into the subchondral bone show some signs of repair with the formation of neocartilage [1], probably due to the infiltration of the defect with bone marrow-derived stem cells from the underlying subchondral bone [2]. This principal is employed in the surgical technique of subchondral drilling and microfracture to stimulate cartilage repair. However, this can result in the formation of fibrocartilage with properties mechanically inferior to articular hyaline cartilage [3]. Autologous chondrocytes harvested from low-weight-bearing areas of articular cartilage and expanded *ex vivo *are being used for the repair of focal hyaline cartilage defects [4], but evidence suggests that this may fail to halt progression of degenerative changes in the joint [5].

There has been a recent interest in cell-based therapies for cartilage repair using adult stem cells or undifferentiated progenitor cells. Stem cells have been reported to be present in many adult human tissue types, including bone marrow, subcutaneous adipose tissue, and the infrapatellar fat pad (IPFP) [6-9]. Compared with bone marrow, IPFP is reported to give a higher yield of stem cells and there is reduced pain and morbidity associated with the harvest of cells [8]. In preliminary work, we identified perivascular cells in the IPFP tissue which stained with a monoclonal antibody, 3G5 [10]. The antigen recognised by 3G5 is a cell surface ganglioside characteristic of retinal vascular pericytes, which have been shown to have multidifferentiation potential [11-15]. It has been suggested that, if distributed widely with vascular capillaries, pericytes may account for stem cells in other tissues [16-18]. In support of this theory, a subendothelial network of pericyte-like cells has been identified using 3G5 in the vascular bed in many human tissues [19], and indeed many of the tissues from which stem cells have been isolated have good vascularisation. A minor population of bone marrow-derived mesenchymal stem cells has also been found to be positive for 3G5 [20].

The defining properties of stem cells are self-renewal and multipotency. Unfortunately, these crucial properties in adult stem cells show donor variability and may become limited on expansion in monolayer culture [21,22]. As expansion is invariably needed to increase the cell number for clinical applications, it is important to achieve expansion without a significant compromise of differentiation potential. Fibroblast growth factor-2 (FGF-2) is a potent mitogen for a variety of cell types derived from the mesoderm, including chondrocytes [23,24]. It has been shown to enhance proliferation and differentiation of bone marrow-derived stem cells [25-28]. FGF produces diverse and sometimes paradoxical effects on cell proliferation and differentiation which are cell-type-dependent [29]. This highlights the need for caution in extrapolating the effects of FGF-2 from one cell type to another. We have previously shown that IPFP-derived cells are able to undergo chondrogenic differentiation [30], but the effect of FGF-2 on the expansion and subsequent chondrogenesis in these cells has not been previously investigated.

In our investigation of the potential of IPFP-derived cells from elderly osteoarthritic patients undergoing joint replacement, we characterised the cells and investigated the chondrogenic response to expansion in FGF-2 in chondrogenic cultures. To further explore the cell surface characterisation, single cells were clonally expanded and stained for a panel of stem cell markers, including 3G5. To allow for the effect of inherent variability in the differentiation potential of cells between individuals [31], we carried out a patient-matched comparison of the chondrogenic potential of cells expanded with and without FGF-2.

## Materials and methods

The IPFP was obtained with ethical approval and fully informed consent from six patients undergoing total knee replacement for osteoarthritis.

### Immunohistochemical staining of tissue sections and cell aggregates

The IPFP tissue and cell aggregates were fixed for 2 hours in 4% formaldehyde (BDH Ltd, Poole, UK)/Dulbecco's phosphate-buffered solution (DPBS) (Cambrex, Wokingham, UK). The samples were then washed in 70% industrial methylated spirit (BDH Ltd) and placed in a Shandon Citadel 2000 tissue processor (Thermo Electron Corporation, Runcorn, UK). Paraffin-embedded sections (5 μm) were taken and mounted on slides precoated with Superfrost Plus (Menzel Glaser GmbH, Braunschweig, Germany), dried in air, and left at 37°C overnight. All incubations were performed in a humidity chamber at 20°C to 21°C, and all washes and dilutions were done in DPBS unless otherwise stated.

#### 3G5 staining of tissue sections

The slides were placed in 0.01 mmol citrate buffer (BDH Ltd) for 10 minutes in a microwave at mid-power followed by cooling to 30°C on ice. Sections were immunostained for 1 hour in undiluted mouse anti-3G5 IgM prepared from a 3G5 hydridoma line (courtesy of Ann Canfield, University of Manchester, UK) followed by washing and incubation for 1 hour in rabbit anti-mouse biotin-conjugated secondary antibody (1:40 with 1% bovine serum albumin [BSA]; Dako, Ely, UK). Mouse IgG antibody was used as a control (Santa Cruz Biotechnology, Santa Cruz, CA, USA). Endogenous peroxidase activity was quenched for 5 minutes with 3% hydrogen peroxide (Sigma-Aldrich, Poole, UK) in methanol (BDH Ltd). Nonspecific binding was blocked with 10% normal rabbit serum (Sigma-Aldrich) diluted in 1% BSA for 1 hour.

#### Alpha smooth muscle actin staining of tissue sections

Wash 1 was made up with 500 mL DPBS, 0.15 M NaCl, and 0.5% BSA, and wash 2 was made up with 500 mL DPBS, 0.15 M NaCl, and 0.1% BSA. Sections were immunostained for 1 hour in mouse anti-human alpha smooth muscle actin (αSMA) (1:400 in wash 1; courtesy of A. Canfield) followed by washing in wash 1 for 1 hour and incubation for 1 hour in rabbit anti-mouse biotin-conjugated secondary antibody (1:50 in wash 1). Mouse IgG antibody was used as a control. The slides were then placed in wash 2 for 1 hour. Endogenous peroxidase activity was quenched for 30 minutes by placing the slides in wash 1.

#### von Willebrand factor staining of tissue sections

Blocking solution was made up with 20% normal donkey serum (Sigma-Aldrich). Sections were immunostained for 1 hour in serum-protein-absorbed rabbit anti-human von Willebrand factor (vWF) IgG (1:250 with 0.1% BSA in blocking solution; Dako) followed by washing and incubation for 1 hour in donkey anti-rabbit biotin-conjugated antibody (1:300 with 0.1% BSA in blocking solution; Dako). Rabbit IgG was used as a control (Santa Cruz Biotechnology). Endogenous peroxidase activity was quenched for 30 minutes with 0.3% hydrogen peroxide in methanol. Nonspecific binding was blocked for 10 minutes with the blocking solution.

#### Collagen type I, type II, and aggrecan staining of cell aggregate sections

Sections were preincubated at 37°C with 0.1 U/mL chondroitinase ABC (Sigma-Aldrich) for 1 hour and then immunostained for 16 hours at 4°C with goat anti-human collagen type I (C-18 polyclonal), collagen type II (N-19 polyclonal) (both from Santa Cruz Biotechnology), or rabbit anti-human aggrecan (BR1) (all at 1:100 dilution) followed by washing and incubation for 30 minutes in donkey anti-goat IgG biotin-conjugated secondary antibody (Santa Cruz Biotechnology) for collagen type I and collagen type II and donkey anti-rabbit IgG biotin-conjugated secondary antibody for aggrecan (all at 1:250 dilution). Goat IgG antibody (Santa Cruz Biotechnology) was used as a control for collagen, and rabbit IgG was used as a control for aggrecan. Endogenous peroxidase activity was quenched for 5 minutes with 3% hydrogen peroxide in methanol. Nonspecific binding was blocked for 1 hour with 10% normal donkey serum diluted in 1% BSA.

For visualisation, sections were incubated for 30 minutes in streptavidin-peroxidase complex (1:500; Dako), rinsed in distilled water, and incubated in fast-DAB (3,3'-diaminobenzidine) peroxidase substrate (Sigma-Aldrich) for 5 minutes and counterstained in diluted filtered haematoxylin (Sigma-Aldrich) for 15 seconds. Images were then taken with an Axioplan 2 microscope with the use of an Axiocam HRc camera and AxioVision 4.3 software (all from Carl Zeiss Ltd, Welwyn Garden City, UK).

### Cell isolation and culture

The IPFP tissue was dissected and cells were isolated by digestion with 0.2% (vol/vol) collagenase I (Invitrogen, Paisley, UK) for 3 hours at 37°C with constant agitation. The released cells were sieved (70-μm mesh) and washed in basic medium, namely Dulbecco's modified Eagle's medium supplemented with 20% (vol/vol) foetal calf serum, 100 U/mL penicillin, and 100 μg/mL streptomycin (all from Cambrex), with L-glutamine (2 mM). The stromal cells were separated from the adipocytes (floating) by centrifugation at 300 *g *for 5 minutes and were counted and plated at 100,000 cells per square centimetre in monolayer culture in basic medium with and without 10 ng/mL rhFGF-2 (Sigma-Aldrich) supplementation. Cultures were maintained at 37°C with 5% CO_2 _and normal oxygen (20%). Cultured cells from passage 2 were used for cell proliferation rate studies, cell surface epitope characterisation, and cell aggregate culture.

### Cell proliferation rates

Cell proliferation rates were measured for passage 2 cells plated with and without FGF-2-supplemented medium at 10,000 cells per square centimetre in a six-well plate. Cells were trypsinised and collected at days 2, 4, 6, 8, and 10 after plating, and the cell number was determined by counting with a haemacytometer. The viability of the cells was determined by staining with Trypan blue.

### Isolation of clonal populations

Clonal cell populations were derived from single cells obtained by limiting dilution. Freshly isolated cells obtained from a single mixed parent IPFP population (mixed parent population is the original, supposedly heterogenous, population of cells from which the clonal cell lines were derived) were plated at a density of 0.33 cells per well in two polystyrene 96-well flat-bottomed cell culture microplates (Corning Inc., supplied through Fisher Scientific, Loughborough, UK). Based on Poisson distribution statistics, the probability of a clonal population being derived from a single cell at this density is greater than 95% [32]. Thirteen wells where a single cell had been noted initially were identified, and the cell progressed to form a single colony. These colonies were selected as they were thought to arise from a single cell. Wells containing more than one colony were excluded. The selected cell populations were trypsinised on confluence and serially plated in a well of a six-well plate (9.6 cm^2^), a T75 cell culture flask (75 cm^2^), and later a T225 cell culture flask (225 cm^2^) (all from Corning Inc.). Only 4 of these 13 expandable clones reached confluence in T225 flasks. The remaining cells from the mixed parent IPFP-derived population were plated at a concentration of 100,000 cells per square centimetre in a T75 flask followed by a T225 flask on confluence.

### Cell surface epitope characterisation

Confluent passage 2 cells expanded with and without FGF-2, and the four clonal and mixed parent populations were stained with a panel of antibodies for cell surface epitopes. This included antibodies against the following: CD13 (aminopeptidase N), CD44 (hyaluronan receptor), CD90 (Thy-1), LNGFR (low-affinity nerve growth factor receptor), STRO1 (marker for bone marrow-derived stem cell), and CD56 (neural cell adhesion molecule, NCAM) from BD Biosciences (Oxford, UK); CD29 (β1 integrin), CD105 (SH2 or endoglin), and CD34 (marker for haematopoetic cells) from Dako; and 3G5 (marker for vascular pericytes). The cells were incubated for 1 hour with the primary mouse antibodies (undiluted 3G5 and 1:100 dilution for others) followed by fluorescein isothiocyanate-conjugated anti-mouse IgM secondary antibody (1:40 dilution; Dako). For controls, nonspecific monoclonal mouse IgG antibody was substituted for the primary antibody. The cells were incubated with 4',6-diamidino-2-phenylindole stain (1:100 dilution) for 5 minutes, and images were captured with an Axioplan 2 microscope using an Axiocam HRc camera and AxioVision 4.3 software.

### Cell aggregate culture

Three-dimensional cell aggregates (500,000 cells [33]) were cultured at 37°C in 1 mL of chondrogenic media for 14 days (medium changed every 2 days) in a normoxic humidified environment. The chondrogenic culture media contained basic media (as above, but without serum) with 1 × insulin-transferrin-selenium supplement (ITS+1; final concentration 10 μg/mL bovine insulin, 5.5 μg/mL transferrin, 5 ng/mL sodium selenite, 4.7 μg/mL linoleic acid, and 0.5 mg/mL BSA), 37.5 μg/mL ascorbate 2-phosphate, 100 nM dexamethasone, 10 ng/mL transforming growth factor (TGF)-β3, and 100 ng/mL insulin-like growth factor-1 (all from Sigma-Aldrich).

### Gene expression analysis

Quantitative real-time gene expression analysis was performed for the following: aggrecan, versican, perlecan, collagen type I (COL1A2), collagen type II (COL2A1), collagen type IX (COL9A1), collagen type X (COL10A1), collagen type XI (COL11A2), L-SOX5, SOX6, and SOX9. Total RNA was extracted with Tri Reagent (Sigma-Aldrich) from passage 2 cells in monolayer and from cell aggregates at 14 days which had been ground with Molecular Grinding Resin (Geno Technology Inc., St. Louis, MO, USA). cDNA was generated from 10 to 100 ng of total RNA by using reverse transcription followed by poly(A) polymerase chain reaction (PCR) global amplification [34]. Globally amplified cDNAs were diluted 1:1,000 and a 1-μL aliquot of the diluted cDNA was amplified by quantitative real-time PCR in a final reaction volume of 25 μL by using an MJ Research Opticon with an SYBR Green Core Kit (Eugentec, Seraing, Belgium). Gene-specific primers were designed within 300 base pairs of the 3' region of the relevant gene with the use of ABI Primer Express software (Applied Biosystems, Foster City, CA, USA). Gene expression analyses were performed relative to β-actin and calculated using the 2^-ΔΔ*Ct *^method [35]. All primers (Invitrogen) were based on human sequences: aggrecan, 5'-AGGGCGAGTGGAATGATGTT-3' (forward) and 5'-GGTGGCTGTGCCCTTTTTAC-3' (reverse); β-actin, 5'-AAGCCACCCCACTTCTCTCTAA-3' (forward) and 5'-AATGCTATCACCTCCCCTGTGT-3' (reverse); COL1A2, 5'-TTGCCCAAAGTTGTCCTCTTCT-3' (forward) and 5'-AGCTTCTGTGGAACCATGGAA-3' (reverse); COL2A1, 5'-CTGCAAAATAAAATCTCGGTGTTCT-3' (forward) and 5'-GGGCATTTGACTCACACCAGT-3' (reverse); COL9A1, 5'-CGGTTTGCCAGGAGCTATAGG-3' (forward) and 5'-TCTCGGCCATTTTTCCCATA-3' (reverse); COL10A1, 5'-TACCTTGTGCCTCCCATTCAA-3' (forward) and 5'-TACAGTACAGTGCATAAATAAATAATATATCTCCA-3' (reverse); COL11A2, 5'-CCTGAGCCACTGAGTATGTTCATT-3' (forward) and 5'-TTGCAGGATCAGGGAAAGTGA-3' (reverse); L-SOX5, 5'-GAATGTGATGGGACTGCTTATGTAGA-3' (forward) and 5'-GCATTTATTTGTACAGGCCCTACAA-3' (reverse); SOX6, 5'-CACCAGATATCGACAGAGTGGTCTT-3' (forward) and 5'-CAGGGTTAAAGGCAAAGGGATAA-3' (reverse); SOX9, 5'-CTTTGGTTTGTGTTCGTGTTTTG-3' (forward) and 5'-AGAGAAAGAAAAAGGGAAAGGTAAGTTT-3' (reverse); and versican, 5'-TGCTAAAGGCTGCGAATGG-3' (forward) and 5'-AAAAAGGAATGCAGCAAAGAAGA-3' (reverse).

### DNA and glycosaminoglycan assays

The wet mass of cell aggregates was recorded at 14 days and the aggregates were digested overnight at 60°C in 20 μL of 10 U/mL papain (Sigma-Aldrich), 0.1 M sodium acetate, 2.4 mM EDTA (ethylenediaminetetraacetic acid), and 5 mM L-cysteine at pH 5.8. DNA in the papain digest was measured with PicoGreen (Invitrogen) with standard double-stranded DNA (Invitrogen), and sulphated glycosoaminoglycan (GAG) was assayed with 1,9-dimethylmethylene blue (Sigma-Aldrich) with shark chondroitin sulphate (Sigma-Aldrich) as standard [33,36].

### Statistical analysis

Experiments were performed separately with cells from six patients and all experiments were in triplicate. Cell proliferation data, gene expression data, wet mass, GAG assay, and GAG per DNA results are presented as a mean and standard error of the mean. Student paired *t *test and a one-way analysis of variance followed by Bonferroni correction were used to analyse the results from two and four culture conditions, respectively, and determine the level of significance. Statistical analyses were conducted with SPSS statistical software (version 11.5) (SPSS Inc., Chicago, IL, USA). Significance was set at a *P *value of less than 0.05.

## Results

### Immunohistochemical staining of the vasculature in infrapatellar fat pad

**Figure 1:**
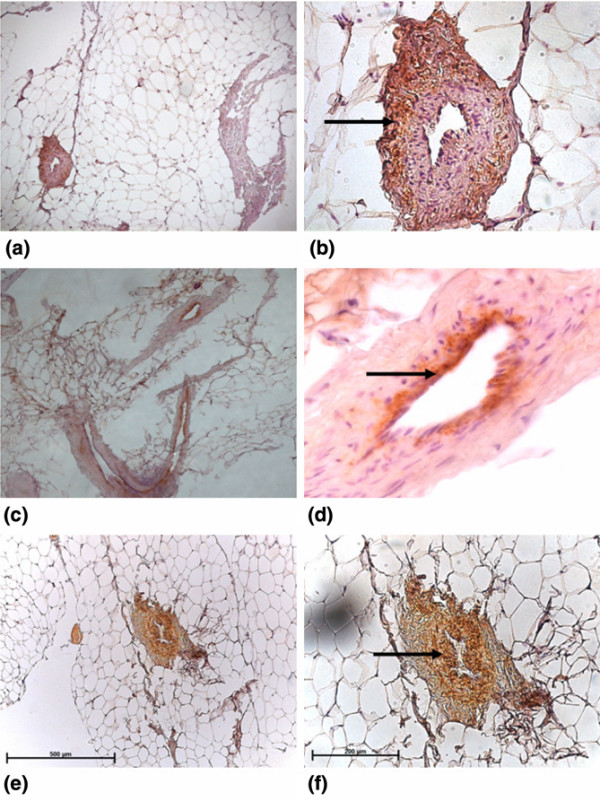
3G5, von Willebrand factor (vWF), and alpha smooth muscle actin (αSMA) staining in the infrapatellar fat pad (IPFP) tissue vasculature. 3G5 **(a, b) **staining predominantly the tunica adventitia consisting of supporting tissue in the vasculature, vWF **(c, d) **staining predominantly the tunica intima consisting of the endothelial layer and the basement membrane, and αSMA **(e, f) **staining predominantly the tunica media consisting of the muscular layer of the arteriole are shown at × 10 (left panels) and × 40 (right panels) magnifications in the IPFP tissue.

IPFP tissue contained large areas of fat-rich adipocytes permeated by a vascular bed of arterioles, venules, and capillaries, which were easily identified in the histological sections. The antibody recognising vascular pericytes, 3G5, predominantly stained cells in the tunica adventitia, which formed the supporting layer of the arterioles (Figure 1a,b), whereas anti-vWF (endothelial cell marker) stained endothelial cells in the tunica intima (Figure 1c,d) and anti-αSMA (smooth muscle cell marker) stained cells in the tunica media, forming the muscular wall of the arteriole (Figure 1e,f). All three antibodies were therefore localised to cells in different regions of the small arterioles. The positive staining for 3G5 in the perivascular cells suggested the presence of pericytes in the IPFP tissue.

### Cell isolation and expansion

**Figure 2:**
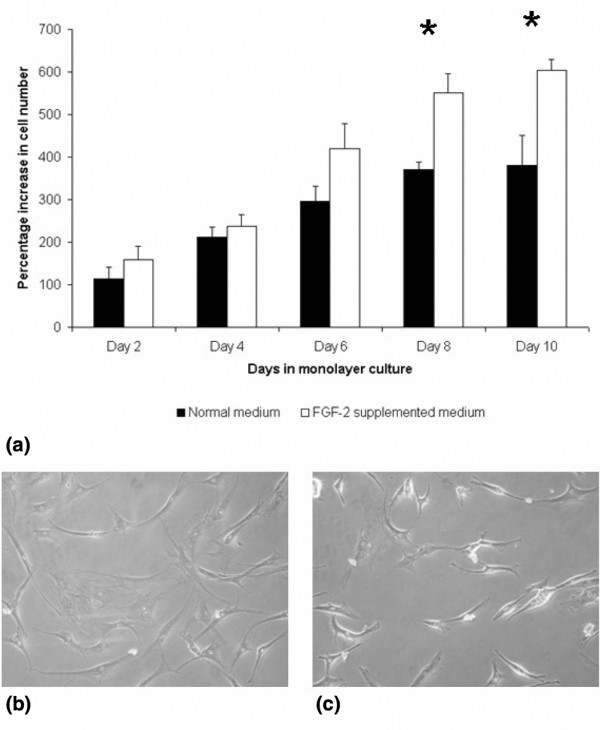
Effects of fibroblast growth factor-2 (FGF-2) expansion on cell proliferation rates and morphology. **(a) **Cell proliferation rates for passage 2 infrapatellar fat pad-derived cells expanded in normal medium (black bars) and FGF-2-supplemented medium (white bars) at days 2, 4, 6, 8, and 10 are shown. Data are mean ± standard error of the mean (n = 6). *P <0.05 (Student paired *t *test). Phase-contrast microscopy of cells expanded in normal **(b) **and FGF-2-supplemented **(c) **media shows that the latter were smaller, more fibroblastic, and less flattened.

Typically, the dissected IPFP tissue from one patient weighed about 20 g, from which 5 g was usually taken to isolate 7.5 million cells. Many of these died in early culture but others attached and proliferated, and at 10 days it was clear that the cells expanded with FGF-2 proliferated more rapidly to give 1.6 times more cells than those without FGF-2 (Figure 2a). Passage 2 flasks without FGF-2 contained 8.6 ± 1.6 million cells, and flasks expanded with FGF-2 contained 13.6 ± 0.5 million cells (*P *= 0.02). The proliferation rate of cells without FGF-2 was 0.13 ± 0.02 doublings per day, and with FGF-2 it was 0.18 ± 0.01 doublings per day (*P *= 0.04). In spite of the faster growth rate, the cells with FGF-2 did not become confluent earlier than the control flasks, which appeared to be due to the smaller size of the FGF-2-supplemented cells (Figure 2b,c). These results appeared to be comparable to those of Wickham and colleagues [9] (2003), who reported 10 to 30 mL of tissue yielding 20 to 35 million cells after two passages.

### Surface epitope characterisation of infrapatellar fat pad cells

**Figure 3:**
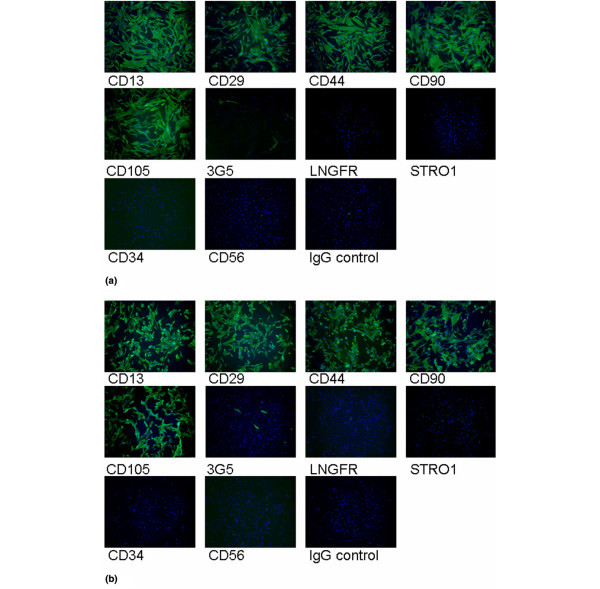
Cell surface characterisation of infrapatellar fat pad (IPFP) cells. Cell surface staining on passage 2 IPFP cells expanded in the absence **(a) **and presence **(b) **of fibroblast growth factor-2 (FGF-2) was performed using a panel of antibodies and fluorescein isothiocyanate-conjugated secondary antibody (green) and DAPI (4'-6-diamidino-2-phenylindole) (blue). Results showed strong staining for CD13, CD29, CD44, CD90, and CD105, weak staining for 3G5, and negative staining for LNGFR, STRO1, CD34, CD56, and the IgG control. The FGF-2-expanded cells are morphologically different from cells expanded in the absence of FGF-2 but show a similar cell surface expression.

IPFP cells at passage 2 expanded with and without FGF-2 stained strongly for CD13, CD44, CD90, and CD105 (markers for mesenchymal stem cells) and for CD29 (β1 integrin) (Figure 3). The cells stained poorly for LNGFR and STRO1, which are markers on freshly isolated bone marrow-derived stem cells, and stained sparsely for 3G5, the marker for vascular pericytes. Staining for the haematopoetic cell marker CD34 and for the neural marker CD56 (NCAM) was negative. This pattern of cell surface staining showed the IPFP cell population to be fairly homogeneous and to express a group of epitopes commonly found on other adult stem cells.

### Clonally expanded infrapatellar fat pad cells

**Figure 4:**
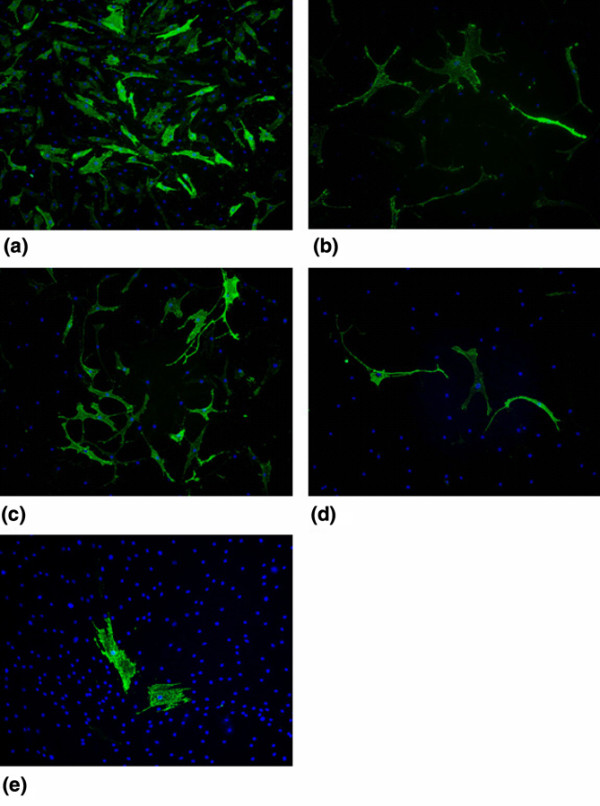
Cell surface characterisation for 3G5 in clonally expanded infrapatellar fat pad (IPFP) cells. Cell surface staining of four clonally expanded IPFP cells **(a-d) **and the parent IPFP population **(e) **using 3G5 and fluorescein isothiocyanate-conjugated secondary antibody (green) and DAPI (4'-6-diamidino-2-phenylindole) (blue) is shown. Results show a heterogenous expression of 3G5 in the mixed IPFP population and also in the clonal IPFP cells.

Freshly isolated IPFP cells were cultured at clonal densities, and four selected clones survived expansion to beyond 20 cell doublings. These cells retained cell surface staining similar to the original parent population, with consistent staining for the various markers identified above (data not shown). The staining for 3G5 was very characteristic as, even in the clonally expanded cells, the proportion of cells positive for 3G5 varied between 1% and 20% (Figure 4). This suggested that the conditions in monolayer culture did not favour 3G5 epitope expression.

### Effect of fibroblast growth factor-2 expansion on subsequent chondrogenic differentiation

**Figure 5:**
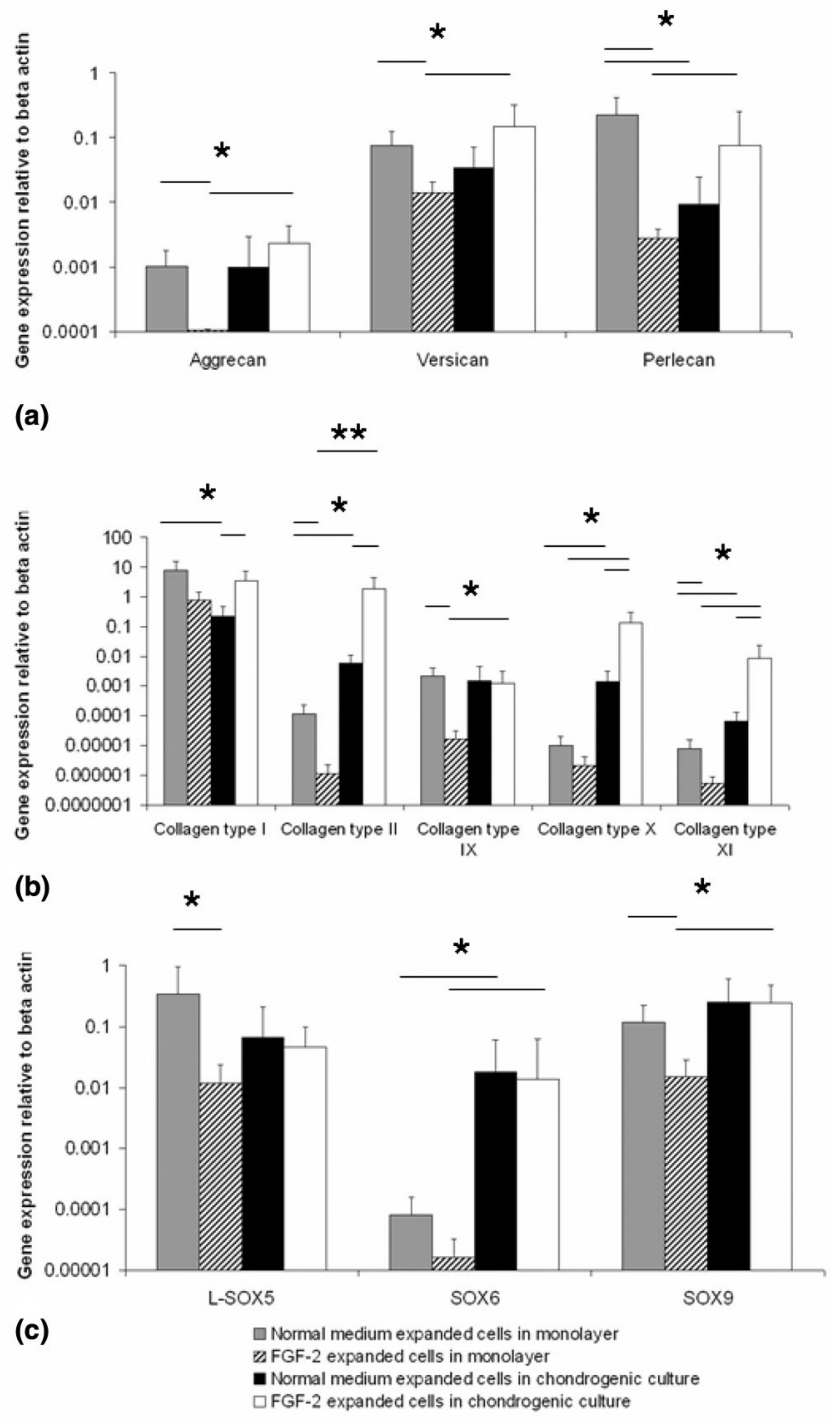
Gene expression in chondrogenic cultures of infrapatellar fat pad (IPFP) cells. Relative gene expression for proteoglycans **(a)**, collagens **(b)**, and SOX genes **(c) **in monolayer with and without FGF-2-supplemented medium to determine basal levels and following subsequent chondrogenesis for 14 days is shown. Data are mean ± standard error of the mean (n = 6). *P < 0.05, **P < 0.001 (analysis of variance with Bonferroni correction).

**Figure 6:**
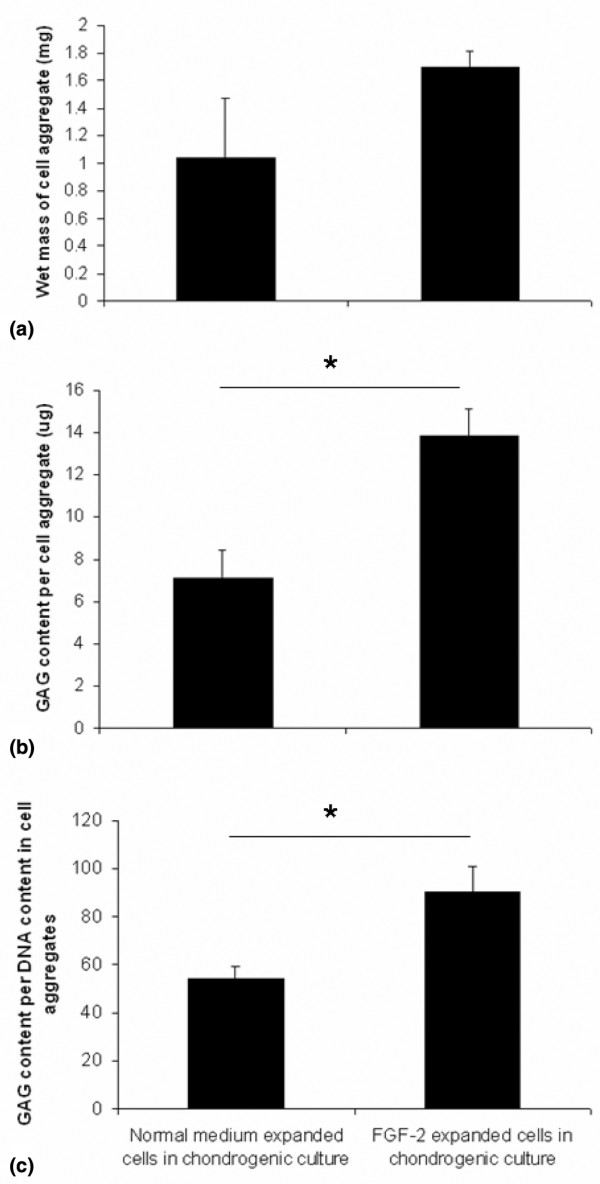
Chondrogenic cultures of infrapatellar fat pad (IPFP) cells and effects of fibroblast growth factor-2 (FGF-2) expansion. Wet weight **(a)**, glycosoaminoglycan (GAG) content **(b)**, and GAG per DNA **(c) **per cell aggregate in chondrogenic cultures after 14 days are shown. Data are mean ± standard error of the mean (n = 6). *P < 0.05 (Student paired *t *test).

**Figure 7:**
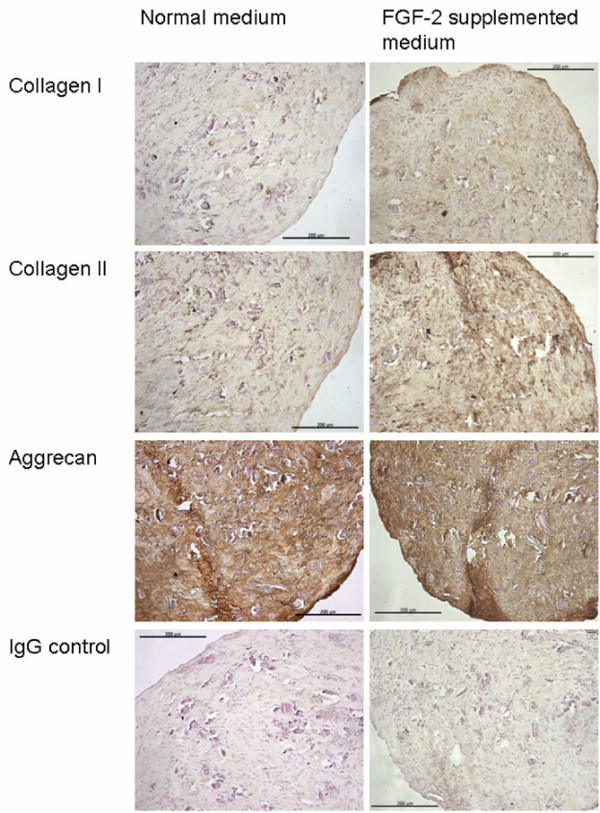
Immunohistochemistry of chondrogenic cultures of infrapatellar fat pad (IPFP) cells. Immunohistochemical staining for collagen type I and II, aggrecan, and control IgG in cell aggregates following chondrogenic differentiation for 14 days in IPFP cells expanded with and without fibroblast growth factor-2 (FGF-2)-supplemented medium is shown.

In monolayer culture, the expression of genes characteristic of chondrocytes, such as aggrecan, collagen type II, IX, and XI, SOX5, and SOX9, was significantly lower in cells expanded with FGF-2 compared with those without (*P *< 0.05) (Figure 5). On subsequent chondrogenic culture, cells expanded with or without FGF-2 showed a chondrogenic response with increased levels of the chondrogenic genes (*P *< 0.05). However, the cells expanded with FGF-2 showed greater increases in gene expression for collagen type I, II, X, and XI compared with cells expanded without FGF-2 (*P *< 0.05). The chondrogenic cultures showed that the cell aggregates from the FGF-2-expanded cells were heavier (Figure 6a) and the GAG content (13.9 ± 1.2 μg) was twofold greater than the non-FGF-2 controls (7.1 ± 1.3 μg) (*P *= 0.01) (Figure 6b). The GAG per DNA ratios were also higher for the FGF-2-expanded cells (*P *= 0.02) (Figure 6c). Immunohistochemical analysis showed significant production of cartilage-like matrix, including collagen type II and aggrecan, in all cell aggregates placed in chondrogenic medium for 14 days, whether expanded in FGF-2 or not (Figure 7). Staining for collagen type I and II and aggrecan was slightly more enhanced for cells expanded in the presence of FGF-2. Although cell aggregates derived from cells expanded in the presence of FGF-2 stained for collagen type I, the immunostaining was increased at the peripheries and was less homogeneously distributed than for collagen type II or aggrecan.

## Discussion

### Cell culture and characterisation of infrapatellar fat pad-derived cells

The rate of proliferation of the IPFP-derived cells in monolayer culture was significantly increased by FGF-2. A comparison of their proliferation rate with other studies is difficult as the only previous study plated cells at lower densities than those used here (10,000 cells per square centimetre [37]) and it was shown that the proliferation rate varied with cell density. No previous study has reported cell surface staining of IPFP-derived cells. It was therefore interesting that they showed a pattern of expression on a high proportion of the IPFP-derived cells and of epitopes commonly abundant on adult stem cells derived from bone marrow and other tissues [20,21,38,39] and that this expression was unaffected by FGF-2 and was maintained in extended culture.

The pericyte marker 3G5 showed a consistent pattern of expression as it was only ever present on a small proportion of cells (typically less than 20%). As this was true even on the progeny derived from a clonally expanded single cell, it suggested that it did not reflect heterogeneity in the cell population but was an epitope expressed by all cells, but only during part of the cell cycle. It is thus possible that IPFP-derived cells were a homogenously 3G5-positive population but that the signals required for consistent expression of 3G5 were absent from monolayer culture. It has previously been noted that the expression of the 3G5 ganglioside varies in culture [40]. The pattern of 3G5 expression has some similarities with STRO1 and LNFGR expression on bone marrow-derived stem cells, which are positive when 'fresh' but become negative with further culture [41-44]. Another possibility is that the expression of 3G5 could be due to culture conditions and not the reminiscence and the demonstration of a cell origin, and further work is needed before any firm conclusions are drawn. The pattern of expression of CD13, CD29, CD44, CD90, and CD105 was consistent during the initial culture on plastic and with passage, suggesting a fairly homogenous population of cells. The effects of FGF-2 were interesting as, although FGF-2 resulted in altered morphological appearance, the cell surface epitope characterisation remained unaltered.

### Clonal IPFP-derived cells retained the cell surface characteristics of the parent IPFP cells, which were similar to mesenchymal stem cells

The clonal populations of IPFP-derived cells appeared phenotypically homogenous and expressed a cell surface epitope profile similar to that of the parent population. It was also notable that the clonal cells continued to express these markers during a long period of cell expansion in culture involving at least 20 cell doublings. The results showed that primary cultures from IPFP-derived cells contained cells that can be grown as clones after limiting dilution and that some clonally expanded cells had high proliferative potential. The lack of CD34 and CD56 expression suggested that none of the clonal cell lines was derived from haematopoetic, neural, or myogenic progenitors or stem cells.

### Evidence for pericytes in the IPFP tissue and IPFP-derived cells

3G5 distinctively stains pericytes and these cells have been shown to have multidifferentiation potential [14]. Histological analyses showed that the IPFP tissue was well vascularised and 3G5 stained cells around small blood vessels but not endothelial cells or smooth muscle cells in sections of the IPFP. These results provided *prima facia *evidence in support of the hypothesis that cells comparable to vascular pericytes were present in the IPFP tissue.

### Chondrogenic differentiation of infrapatellar fat pad cells

The *in vitro *chondrogenic differentiation in IPFP-derived cells has not previously been analysed using quantitative RT-PCR [8,9,37]. This revealed the massive induction of gene expression in going from monolayer culture through chondrogenic differentiation in cell aggregates. It was not surprising to see increased gene expression for collagen type X in chondrogenic culture as the presence of TGF-β in cell culture media has previously been associated with increased collagen type X expression in mesenchymal stem cells [45]. This occurred despite the fact that TGF-β inhibits the terminal differentiation of chondrocytes *in vivo *[46].

FGF-2-supplemented expansion potentiated subsequent chondrogenic differentiation as the FGF-2-expanded cells showed a much greater increase in type II collagen expression than non-FGF-2-expanded cells. Previous studies on *in vitro *cartilage formation have resulted in tissue of low collagen content [47]. The use of FGF-2 may therefore be of particular benefit in increasing the production of matrix in cartilage tissue-engineered *in vitro*. The inhibition of actin stress fibres by FGF-2 in adult human chondrocytes results in an upregulation of SOX9 (S.R. Tew and T.E. Hardingham, unpublished data) and, although there was no direct effect of FGF-2 expansion on SOX9 expression in IPFP-derived cells in monolayer, it may have suppressed subsequent actin stress fibre formation during chondrogenesis.

Chondrogenic differentiation resulted in an increase in total GAG and a greater GAG per DNA ratio in the cell aggregates formed from cells cultured with FGF-2. This is comparable to reports of the effects of FGF-2 on bone marrow-derived mesenchymal stem cells [25-27] and has important implications for the role of FGF-2 in tissue engineering applications of these cells. There was some decrease in total DNA in the chondrogenic cultures, which was previously reported during *in vitro *chodrogenesis in mesenchymal stem cells [48]. FGF-2 is routinely used in the culture of bone marrow-derived mesenchymal stem cells, and we have determined a baseline for the use of FGF-2 in the culture of fat pad-derived cells.

## Conclusion

The present study showed that IPFP tissue contained cells that expressed markers in common with other mesenchymal stem cell markers. The study suggested that pericytes are candidate stem cells in human IPFP tissue, but further work is needed to determine the true origin of these cells. Expansion of these cells with FGF-2 has important consequences for facilitating their chondrogenic differentiation.

## Abbreviations

αSMA = alpha smooth muscle actin; BSA = bovine serum albumin; DPBS = Dulbecco's phosphate-buffered solution; FGF-2 = fibroblast growth factor-2; GAG = glycosoaminoglycan; IPFP = infrapatellar fat pad; LNGFR = low-affinity nerve growth factor receptor; NCAM = neural cell adhesion molecule; PCR = polymerase chain reaction; TGF = transforming growth factor; vWF = von Willebrand factor.

## Competing interests

The authors declare that they have no competing interests.

## Authors' contributions

WSK conceived, designed, and performed the experiments described in this study, was responsible for tissue procurement and processing, and produced the initial version of this manuscript. SRT and ABA helped perform the gene expression analyses. TEH supervised and oversaw the experiments and writing of this manuscript. All authors read and approved the final manuscript.

## References

[B1] Newman A (1998). Articular cartilage repair. Am J Sports Med.

[B2] Shapiro F, Koide S, Glimcher MJ (1993). Cell origin and differentiation in the repair of full thickness defects of articular cartilage. J Bone Joint Surg Am.

[B3] Hunziker EB (2001). Articular cartilage repair: basic science and clinical progress; a review of the current status and prospects. Osteoarthritis Cartilage.

[B4] Brittberg M, Lindahl A, Nilsson C, Isaksson O, Patterson L (1994). Treatment of deep cartilage defects in the knee with autologous chondrocyte transplantation. N Engl J Med.

[B5] Lee CR, Grodzinsky AJ, Hsu HP, Martin SD, Spector M (2000). Effects of harvest and selected cartilage repair procedures on the physical and biochemical properties of articular cartilage in the canine knee. J Orthop Res.

[B6] Johnstone B, Hering TM, Caplan AI, Goldberg VM, Yoo JU (1998). *In vitro *chondrogenesis of bone marrow-derived mesenchymal progenitor cells. Exp Cell Res.

[B7] Zuk PA, Zhu M, Mizino H, Huang J, Futrell JW, Katz AJ, Benhaim P, Lorenz HP, Hedrick MH (2001). Multilineage cells from human adipose tissue: implications for cell-based therapies. Tissue Eng.

[B8] Dragoo JL, Samimi B, Zhu M, Hame SL, Thomas BJ, Lieberman JR, Hedrick MH, Benhaim P (2003). Tissue-engineered cartilage and bone using stem cells from human infrapatellar fat pads. J Bone Joint Surg Br.

[B9] Wickham MQ, Erickson GR, Gimble JM, Vail TP, Guilak F (2003). Multipotent stromal cells derived from the infrapatellar fat pad of the knee. Clin Orthop.

[B10] Khan WS, Andrews JG, Hardingham TE (2005). Immunohistochemical staining of the infrapatellar fat pad vasculature with 3G5, alpha smooth muscle actin and von Willebrand factor [abstract]. Int J Exp Path.

[B11] Rhodin JA (1968). Ultrastructure of mammalian venous capillaries, venules, and small collecting veins. J Ultrastruct Res.

[B12] Meyrick B, Fujiwara K, Reid L (1981). Smooth muscle myosin in precursor and mature smooth muscle cells in normal pulmonary arteries and the effect of hypoxia. Exp Lung Res.

[B13] Brighton CT, Lorich DG, Kupcha R, Reilly TM, Jones AR, Woodbury RA (1992). The pericyte as a possible osteoblast progenitor cell. Clin Orthop Relat Res.

[B14] Doherty M, Ashton B, Walsh S, Beresford J, Grant M, Canfield A (1998). Vascular pericytes express osteogenic potential *in vitro *and *in vivo*. J Bone Miner Res.

[B15] Farrington-Rock C, Crofts NJ, Doherty MJ, Ashton BA, Griffin-Jones C, Canfield AE (2004). Chondrogenic and adipogenic potential of microvascular pericytes. Circulation.

[B16] Gronthos S, Franklin DM, Leddy HA, Robey PG, Storms RW, Gimble JM (2001). Surface protein characterisation of human adipose tissue-derived stromal cells. J Cell Physiol.

[B17] Chiou M, Xu Y, Longaker MT (2006). Mitogenic and chondrogenic effects of fibroblast growth factor-2 in adipose-derived mesenchymal cells. Biochem Biophys Res Commun.

[B18] Tare RS, Babister JC, Kanczler J, Oreffo RO (2008). Skeletal stem cells: phenotype, biology and environmental niches informing tissue regeneration. Mol Cell Endocrinol.

[B19] Andreeva ER, Pugach IM, Gordon D, Orekhov AN (1998). Continuous subendothelial network formed by pericyte-like cells in human vascular bed. Tiss Cell.

[B20] Shi S, Gronthos S (2003). Perivascular niche of postnatal mesenchymal stem cells in human bone marrow and dental pulp. J Bone Miner Res.

[B21] Pittenger MF, Mbalaviele G, Black M, Mosca JD, Marshak DR, Koller MR, Palsson BO, Masters JRW (2001). Mesenchymal stem cells. Primary Mesenchymal Cells.

[B22] Cancedda R, Dozin B, Giannoni P, Quarto R (2003). Tissue engineering and cell therapy of cartilage and bone. Matrix Biol.

[B23] Kato Y, Gospodarowicz D (1985). Sulfated proteoglycan synthesis by confluent cultures of rabbit costal chondrocytes grown in the presence of fibroblast growth factor. J Cell Biol.

[B24] Zannettino AC, Paton S, Arthur A, Khor F, Itescu S, Gimble JM, Gronthos S (2008). Multipotential human adipose-derived stromal stem cells exhibit a perivascular phenotype *in vitro *and *in vivo*. J Cell Physiol.

[B25] Martin I, Muraglia A, Campanile G, Cancedda R, Quarto R (1997). Fibroblast growth factor-2 supports *ex vivo *expansion and maintenance of osteogenic precursors from human bone marrow. Endocrinol.

[B26] Bianchi G, Banfi A, Mastrgiacoma M, Notaro R, Luzzatto L, Cancedda R, Quarto R (2003). *Ex vivo *enrichment of mesenchymal cell progenitors by fibroblast growth factor 2. Exp Cell Res.

[B27] Solchaga LA, Penick K, Porter JD, Goldberg VM, Caplan AI, Welter JF (2005). FGF-2 enhances the mitotic and chondrogenic potentials of human adult bone marrow-derived mesenchymal stem cells. J Cell Physiol.

[B28] Rider DA, Dombrowski C, Sawyer AA, Ng GH, Leong D, Hutmacher DW, Nurcombe V, Cool SM (2008). Autocrine fibroblast growth factor 2 increases the multipotentiality of human adipose-derived mesenchymal stem cells. Stem Cells.

[B29] Dailey L, Ambrosetti D, Mansukhani A, Basilico C (2005). Mechanisms underlying differential responses to FGF signaling. Cytokine Growth Factor Rev.

[B30] Khan WS, Adesida AB, Hardingham TE (2007). Hypoxic conditions increase HIF2alpha and enhance chondrogenesis in stem cells from the infrapatellar fat pad of osteoarthritis patients. Arthritis Res Ther.

[B31] Huang JI, Kazmi N, Durbhakula MM, Hering TM, Yoo JU, Johnstone B (2005). Chondrogenic potential of progenitor cells derived from human bone marrow and adipose tissue: a patient matched comparison. J Orthop Res.

[B32] Taswell C (1981). Limiting dilution assays for the determination of immunocompetent cell frequencies. J Immunol.

[B33] Tew SR, Li Y, Pothacharoen P, Tweats LM, Hawkins RE, Hardingham TE (2005). Retroviral transduction with SOX9 enhances re-expression of the chondrocyte phenotype in passaged osteoarthritic human articular chondrocytes. Osteoarthritis Cartilage.

[B34] Al Taher A, Bashein A, Nolar T, Hollingsworth M, Brady G (2000). Global cDNA amplification combined with real-time RT-PCR: accurate quantification of multiple human potassium channel genes at the single cell level. Yeast.

[B35] Livak KJ, Schmittgen TD (2001). Analysis of relative gene expression data using real-time quantitative PCR and the 2(-Delta Delta C(T)) method. Methods.

[B36] Singer VL, Jones LJ, Yue ST, Haugland RP (1997). Characterization of PicoGreen reagent and development of a fluorescence-based solution assay for double-stranded DNA quantitation. Anal Biochem.

[B37] Mochizuki T, Muneta T, Sakaguchi Y, Nimura A, Yokoyama A, Koga H, Sekiya I (2006). Higher chondrogenic potential of fibrous synovium- and adipose synovium-derived cells compared with subcutaneous fat-derived cells. Arthritis Rheum.

[B38] Haynesworth SE, Baber MA, Caplan AI (1992). Cell surface antigens on human marrow derived mesenchymal cells are detected by monoclonal antibodies. Bone.

[B39] Baddoo M, Hill K, Wilkinson R, Gaupp D, Hughes C, Kopen GC, Phinney DG (2003). Characterisation of mesenchymal stem cells isolated from murine bone marrow by negative selection. J Cell Biochem.

[B40] Nayak RC, Berman AB, George KL, Eisenbarth GS, King GL (1988). A monoclonal antibody (3G5) defined ganglioside antigen is expressed on the cell surface of microvascular pericytes. J Exp Med.

[B41] Simmons PJ, Torok-Storb B (1991). Identification of stromal cell precursors in human bone marrow by a novel monoclonal antibody, STRO-1. Blood.

[B42] Bruder SP, Horowitz MC, Mosca JD, Haynesworth SE (1997). Monoclonal antibodies reactive with human osteogenic cell surface antigens. Bone.

[B43] Stewart K, Walsh S, Screen J, Jefferiss CM, Chainey J, Jordan GR, Beresford JN (1999). Further characterisation of cells expressing STRO-1 in cultures of adult human bone marrow stromal cells. J Bone Miner Res.

[B44] Quirici N, Soligo D, Bossolasco P, Servida F, Lumini C, Deliliers GL (2002). Isolation of bone marrow mesenchymal stem cells by anti-nerve growth factor receptor antibodies. Exp Haematol.

[B45] Zuk PA, Zhu M, Ashjian P, De Ugarte DA, Huang JI, Mizino H, Alfonso ZC, Fraser JK, Benhaim P, Hedrick MH (2002). Human adipose tissue is a source of multipotent stem cells. Mol Biol Cell.

[B46] Yang X, Chen L, Xu X, Li C, Huang C, Deng CX (2001). TGF-beta/Smad3 signals repress chondrocyte hypertrophic differentiation and are required for maintaining articular cartilage. J Cell Biol.

[B47] Erickson GR, Gimble JM, Franklin DM, Rice HE, Awad H, Guilak F (2002). Chondrogenic potential of adipose tissue derived stromal cells *in vitro *and *in vivo*. Biochem Biophys Res Commun.

[B48] Sekiya I, Vuorist JT, Larson BL, Prockop DJ (2002). *In vitro *cartilage formation by human adult stem cells from bone marrow stroma defines the sequence of cellular and molecular events during chondrogenesis. Proc Natl Acad Sci USA.

